# Introductory to Lectures on Philosophy of Medicine, in the Spring Course of Rush Med. College, March 8, 1872

**Published:** 1872-05

**Authors:** J. E. O’Brien


A MONTHLY RECORD OF

*Medicine, Surgery and the Collateral Sciences.*

EDITED BY J. ADAMS ALLEN, M.D., LL.D.; AND WALTER HAY, M.D.

---

VOL. XXIX. — MAY, 1872. — NO. 5.

---

### Original Communications.

ARTICLE I. — *Introductory to Lectures on Philosophy of Medicine, in the Spring Course of Rush Medical College, March 8th*, 1872. By J. E. O'BRIEN, M.D.*

*Gentlemen*—It seems as though nature proposed to baptize our young Spring Course this morning; the ruins of our burned city, the ashes of her recent desolation, are lying deeply buried beneath the snow. *Science* casts down to us the crystals of her knowledge just like the snow: if we treasure them properly, they will spring forth in our minds as clearest fountains of knowledge and truth; if we let them lie uncared for where they fall, they will be quickly swept into oblivion.

If I understand the object of the Spring Course correctly, it is to give you additional opportunities for study; and your presence here indicates that you desire to take advantage of the practical

* For the historical part of my subject, I am indebted to the lectures of Prof. Glover, of England.

aids which we propose to give you, so as to make yourselves a little better qualified for physicians than the average graduate. Among all the circumstances which influence a man's career, there is none so powerful as his own *will*, and if you determine to widen your knowledge and enlarge your understanding, success will assuredly attend you. There is vast room for improvement over the mere average acquirements which physicians are asked to have. The fact that you see a majority of the physicians around you settling down to a routine-empirical practice, is the very reason why you should expect it to be wrong.

"Old Rip Van Winkle had a Grandson Rip,"

and

—"Now then Mr. V.,
He's good for *something*, make him an M.D."

This young Rip Van Winkle, M.D., has multiplied exceedingly in town and city and college. Literary colleges are particularly prolific in his species, and he shoals in our profession, expecting that it will make a man of him, when the fact is, that we want *men* to come to us, and make our profession what it should be. Nothing can educate a man unless he *works*. Active contact with the world is the grand incentive to labor, and labor accomplishes all things that are worth learning. It is therefore grateful to see you here, as showing by your presence an intention to work for success in a profession which is worthy of life-long devotion, which traces its brilliant history back to the origin of history itself, and includes among its treasures such minds as those of Hippocrates, Galen, Sydenham, Hunter, Harvey, Bacon, and his disciples, and in our own day, Virchow, Beale, Rokitansky, Dalton, and the rest of the grand army of investigators and philosophers, who think a whole life too short for the service of this great art.

There are no text books on the branch which we propose to consider. What I shall give you will be principally the practical results of my own reasoning on the mechanism of disease and collateral subjects, but wherever I can find any ideas bearing upon the subject, I will appropriate them and work them into my course. You will find my studies strongly tinged with the teachings of our Professors, and I desire to give them the credit at the start, as I

shall probably not know myself how greatly their principles have become incorporated in my memory.

There is one qualification which I apprehend an instructor should have who proposes to teach philosophy; he ought to be a philosopher himself. I would therefore have you understand that I do not pretend to *teach* philosophy to you, but shall be well content if I can assist you in *learning* it. Another difficulty besides the fact that there are no text books is, that I have had little time to think over a plan for our course, having been out of the city a great part of the winter on business for the college, but I have hastily sketched out the following plan, and shall follow it as closely as circumstances will permit.

We will begin with disease as a central figure, and see what philosophy we can find in connection with it. Around this we will group causes influencing disease, and consider in detail the operations of climate, miasma, ochlesis, soil, temperament, habit, idiosyncracy, hereditary influence, hygiene, and so on. Then we will have a chapter on "how nature cures disease," illustrating with particular diseases. Then we will see what philosophy we can find in the operations of the cures of art, as compared with those of nature. I have made a special study of inflammation, congestion, etc., and shall discuss it in the early part of the course. After finishing our consideration of philosophy of medicine, I shall have the privilege of assisting you in the study of diseases of the skin. We shall not study them so much as a specialty, as for practical benefit, for I hold that the true value of a thing is its practical value. The diseases of the skin, then, which we will mostly consider, are those that will occur most frequently in your practice.

I will now give you a definition for philosophy of medicine. It is *reasoning about* medicine. Every physician should be a philosopher to this extent, contrasting directly with those who are content to give medicine in disease, without inquiring into the relations of the disease, or the mechanism of cure. It is a favorite custom with these routinists to prescribe a dozen or so of remedies at once, hoping that "if one misses, some other may hit the mark;" and I have known writers in the journals to seem to glory in this kind of thing. I call it mitrailleuse practice. If they would confine it to their own practice, it might be endured, but when they attempt to teach such gross empiricism to others, they do incalculable mischief to the cause of investigation, and of philosophy. Out of the extreme habit of multi-dosing, some of these physicians have found a short road to entire skepticism of medicine, and another class think that every man may be his own physician.

The class of skeptics who limit the physician's usefulness to the use of specifics, name the very cases when he might be dispensed with, a specific cure requiring no knowledge of anatomy or physiology, and but little knowledge of disease for its administration.

There is but one specific, and Ague is its prophet; (because it reveals the properties of quinia.) The treatment of ague has been reduced to almost mathematical precision, it might, therefore, be left to the care of persons of good judgment other than physicians. It is the only disease that might be so left without danger, and frequently there is danger in it. In nearly all other diseases the watchful care of the physician is necessary to prevent destruction of tissue and impairment of function among the organs of life. In inflammations, which constitute more than two-thirds of all disease, the morbid action, if not controlled by the physician's assistance in aid of nature, will run on to suppuration, adhesion, abscess, destruction of organic tissue, loss of function, and often, death; results varying with the nature of the organ attacked, and its importance to life. In these and most other diseases the educated physician can hasten recovery, preserve the organs and limit mortality, giving drugs, not because they are said to be "good for it," nor because *Dr. Scovendique* uses them, but because he knows the condition of the organs in health and disease, and knows how to produce definite results upon them by the active agents of the materia medica. Recognizing the different stages of disease by a careful interrogation of all the signs exhibited to the closest examination, he uses remedies to assist nature, supporting her where the powers of life fail, using sedative means when inflammatory or other morbid action runs too high.

"The blood goes down to the capillaries of the body, where all the changes of life occur." The educated physician knows the normal constituents of the blood; he knows how many parts of water, of salts, of albumen, of fibrin, of iron, etc., it contains in health; he knows the variations of these constituents in disease, and corrects them; regulates the action of the nervous system, which presides over all the operations of life; knowing the sympathetic connection of organs with each other, he inquires into the condition of each, strengthens those of digestion and blood-making, stimulates those which carry out of the system waste material, if the condition of the system requires it, and puts the diseased part at rest, or acts upon it by certain means to improve its condition and restore its normal functions.

Of the great armory of drugs embraced in our materia medica, only one is a certain cure, and that for only a single disease. The cause of this has been too much attention paid to drugging, and too little time devoted to the study of the human organism, its anatomy and physiology, in health and disease. It is like having a large assortment of fine tools to repair a disabled chronometer without understanding the workings of the latter. If the tools are used promiscuously upon the delicate machinery, the result will be obvious. If they are used without the utmost degree of intelligence, and knowledge as to what is the matter, the result will be not less disastrous. So it is in acting upon the fine mechanism of man: if we do not know how disease affects it, and what it will do with the medicine exhibited to it through the blood, we shall do harm, and not good. If in acute congestion the remedies suited to the latter stages of inflammation are applied, the injury done to diseased organs may last a lifetime, and shorten it. But if the anatomy of the parts is an open book to the physician—if he recognizes the changes going on there under the influence of disease—he will apply the proper remedies to assist nature as required, perhaps oppositely, in the different stages, eliminate morbific matter through the proper emunctories, see that the organs of the body react favorably upon each other and the diseased part, by keeping them all in a healthy state, and, finally, put the diseased organ at rest by compelling others to perform its functions vicariously, for which nature has beautifully provided. All this the intelligent physician can do in the majority of diseases. The instruments are all ready to his hand, and perfect of their kind. If proper care is exercised in securing pure drugs, the materia medica furnishes them in the most active and convenient state.

Who, knowing the good that physicians accomplish who practice under the philosophical principles stated, *i. e.*, uniting materia medica to anatomy, physiology and pathology, can say that the physician may be dispensed with? If he could do no more in disease than one of the duties mentioned, that of putting the diseased organ at rest, he would still be of incalculable service.

The vicarious action of organs is one of the institutes of nature herself. If a kidney cannot perform its function of separating urea from the blood, the intestines will make an effort, though feebly, to eliminate it. The intelligent physician will assist nature in this regard, will stimulate the intestines to increased action upon the morbid matter, thus giving the kidney time to rest, and time to recover under appropriate treatment. In scarlatina, he who knows drugs but not anatomy, stimulates the inflamed kidney to increased action, as does also he who thinks that "Like cures like," giving diuretics until the inflamed organ, already overworked, breaks down, and dropsy is the result. If the mission of the enlightened physician were to correct this abuse alone, the results from the fatal scarlet fever should give him a proud and permanent position.

So much for those who are skeptical of medicine. If they would use the efficient agents of the materia medica upon the principles stated, and with proper knowledge of what is the matter, they could favorably modify the course of most diseases, could limit mortality, and, assisting nature in the cure, leave the recovered organs in vastly better condition than disease in its onward march would, if opposed only by *unaided* nature. The greatest test of skill is not in curious combinations of prescription ingredients, which are already, some of them, the most beautiful and complex products of the chemist's art. The test of skill in medicine is, to know *what is the matter*—such knowledge requiring all the light that the human mind can throw upon the anatomy of man.

It is not *names* that amount to anything. It is knowing the actual changes going on among the vessels, nerves and cells within the diseased organism, under the influence of disease; knowing which, appreciating the actual condition of the parts at present, and anticipating the changes that will occur to-morrow, the mere prescription and combination of powerful remedies resolves itself into simplicity itself, and brings medicine as near the condition of an exact science as it is possible to bring it. If every one shall ever be his own physician, it will be when we shall have much more time for study than at present; but while that time may be more or less remote, all that we may know of ourselves, and how we live and move, should be acceptable to the people, and all that brings them into closer relation with their physicians, should be beneficial to them and to us. They are our judges, and should estimate us by some reasonable rule, such as is used in estimating other men. Success is a tolerably fair criterion—would be more so if we were all agreed as to what success is. If physicians are chosen for their successful cures, they ought to be satisfied. If for their successful fees, there is room for complaint, and we admit a host of quacks, who are usually the most successful financially.

But the true test is knowledge—and, most of all, knowledge of the human machine—in health and disease. And so the tendency of the profession now is, to gratify the growing taste for physiological knowledge among thinking people, and some good writers of the profession, like Huxley of England, are giving to the public the benefit of their care and study. It is the true way to elevate the standard of the profession; the public is the final examining board for physicians, and should have data to judge by. The mystery which physicians of a past age threw around their art made it impossible for people to discriminate between the true and the false. Hence,Empiricism prospered; hence,Humoralism,Solidism, Brunonianism, Hydropathy, Animal Magnetism, The doctrine of Signatures, Homœopathy, The Royal Touch, Phrenology, Metallic Traction, Thompsonism, and a thousand other humbugs, have in turn presented themselves to the attention of mankind and flourished—some owing their existence to the influence of the imagination ; some appealing directly to credulity, and many having some grain of truth in their composition, but soon burying it beneath a mass of "false facts," whenever pecuniary gain threw the necessary inducement in the way of "prophets," stimulating them to draw general conclusions from special premises. (Mystery, however, was not the cause of the immense popularity of a certain nostrum some years since, as related by a London Medical Journal. "Brandy and salt, which was a sovereign cure, as they all are, for all the ills that flesh is heir to. The brandy made the heart glad; the salt increased the thirst for more brandy." No mystery about that—part of the belief endures yet.)

There is an element of truth in all delusions, and many valuable ideas and drugs have been brought to light by the votaries of the sects mentioned. As long as they are specialists in investigation, they may increase the general store of knowledge; it is when they attempt to found principles of practice upon the false premises, that harm is done; the only premises allowed should be anatomy and physiology—in health and disease. Upon this broad platform such sophistry as "*Similia Similibus Curantur*" can never stand.

Knowing the definite results to be produced upon the human organism in order to modify disease, the learned physician selects his remedies for their virtues; rejecting all that is false and injurious, he selects that which is true and good wherever found.

From the time of Hippocrates, 580 years before the Christian era, to our own day, there have been two principal sects in medicine: they are the Theorists and the Empirics, with here and there a true philosopher illuminating by his reason the historical page of our art.

The empirics are those who are content to treat disease without ever asking the important questions *why?* and *how?* Always paying great attention to the symptoms actually presented to them, they have been satisfied with this, and have not attempted to trace the symptoms to their source, nor to ask of nature why they are manifested.

The theorists, on the other hand, take the opposite extreme, and carry medicine off into the domain of speculation, where they soon lose sight of all that is practical or useful.

The true philosopher avoids these extremes. Unlike the empiric, he traces the phenomena of disease back to their causes, and these he pursues as far back as human knowledge extends, studying, as he goes, the *relations* of causes to each other and to the phenomena produced, until he arrives at general laws established by nature. He differs from the theorist because he never cuts loose from *facts*, never extends his reasoning beyond the limits warranted by logical truth, and always keeps in view the practical utility of his labors.

It may be said that the ancients were *compelled* to enter the ranks of one or the other of the sects named, that they had to be either dogmatists or empirics, because they had no facts to establish their premises upon. True, they had no anatomy. Prof. R. was not with them; our gifted demonstrator was among the generations yet unborn; physiology was unknown to them—they knew nothing of the mechanism of respiration or circulation—nothing of the chemistry of digestion—they *had* no chemistry. All they knew was that diseases affected men similarly or differently, that diseases had some rules about duration, and so on. They also learned that some substances would purge, others vomit, and so on. It is true, this was about the extent of their knowledge, but they might still have been philosophers if they had interrogated nature for facts, and had kept their reasoning within the logical limits which require that it shall retain a *connection* with facts.

Hippocrates narrowly escaped being such a philosopher. In symptomatology his teaching is worth reading at this day; but in ætiology his doctrine was no better than the imperfect state of physical science in his time. According to him, there were four elements—earth, air, fire, and water; and in the animal body four humors—blood, phlegm, bile, and atrabile. Diseases were said to depend upon a derangement of the humors, and restoring their equilibrium was what would cure the disease.

There were seven doctors of the Hippocratic family during some three hundred years, medicine being hereditary with the Greek priesthood. The greatest of the family was Hippocrates the *second*, and to him most of the writings of the family are credited. The Hippocratic writers knew little of anatomy, or what we understand by pathology. The same kind of prejudice with regard to dissection existed then as now among the unenlightened Mahometans, and in Hindostan. They drew their anatomical ideas from the analogy of the inferior creation. They did not properly distinguish ligaments, muscles, vessels, and nerves—nevertheless they had *some* notion of the vessels, glands, and interior organs.

As previously noticed, it was in symptomatology of disease that this school particularly shows, and some of their doctrines on this head have a generality which renders them to a certain extent applicable for all time; for instance, the doctrine of the three periods of disease, crudity, coition, crisis, the signs of these three periods were noted with the greatest care—the phenomena which denoted a favorable crisis or a dangerous metastasis were most rigorously determined, and upon all this was founded a system of prognosis which even now may furnish many valuable hints.

As an example of the refinement to which observation had reached at this period, the doctrine of critical days may be cited.

According to this, nature had a tendency to evacuate the morbid matter after the attack, at certain periods in preference to others; these days were the 4th, 7th, 11th, 14th, 17th, and 20th.

From the nature of the Hippocratic theory of disease, great attention was paid to the signs afforded by the urine. All the states of this fluid, and the sediments which can be observed by the unassisted senses, were carefully attended to. In diet, regimen, effects of climate, and so forth, much was done by this school. Most of their remedies were drawn from the vegetable kingdom, with the exception of alum and some preparations of lead and copper. They practiced bleeding, and made great use of purgatives, sudorifics, poultices, and baths. In summing up their merits, we may honor them as models of observers during their age; they carried the greatest care into the practice of their art.

After the time of Hippocrates, the philosophy of medicine slept until the important study of anatomy began to be cultivated. Natural history and anatomy date their existence as sciences from the time of Alexander; and in all probability his extensive conquests and those of his successors, by rendering the Greeks familiar with the natural products of various regions, contributed very much to the circumstance.

Aristotle has described with astonishing accuracy, on the whole, the comparative anatomy of animals, and many of the elements of the frame.

*Theophrastus* was the most distinguished of the naturalists who followed Aristotle. His labors were directed more particularly to the vegetable kingdom, its structure and physiology. He also described several *diseases* of plants, as the ergot of rye.

But the most remarkable observers in the field of natural history and anatomy flourished at Alexandria under the reign of the Ptolemys. Herophilus and Erasistratus deserve special mention. The former carried his researches so far that one of the earlier modern anatomists considered him infallible. He dissected great numbers of human bodies, and even *living criminals*, as related by Celsus.

But the pathology of these great men was not on a level with their anatomy. Erasistratus' modification of the humoral doctrine was but little improvement on Hippocrates. Many of the disciples of the Alexandrian school were nearly as celebrated as their masters.

The Romans did but little for medicine; Celsus was but little more than a compiler. He, however, described surgical operations.

The first attempt at getting materia medica into book form was made by Dioscorides, of Anazarba, who probably lived about the time of Nero. (I thought it best to give you the dates, as you might confuse Anazarba with a little town up in Michi*gan*.) Although Dioscorides was a theorist, and explained the action of remedies by their elementary qualities, still he was a philosopher in the important branch of seeking for and collating facts, and for many centuries his works were the principal in which botany and materia medica were studied. Having traveled in the train of Roman armies, the information which he got together by his surpassing industry, was such that he was for the long time mentioned regarded as the *ne plus ultra* in the department which he professed, and he is probably still venerated as such in the schools of Fez, Ispahan and Bokkhara. Many of his remedies were sufficiently absurd; others still retain their place in the treatment of diseases for which he indicated their use. It is evident from his works how the resources at the disposal of the physician had increased since the time of Hippocrates, and in this fact you will see that philosophy of medicine was *dawning*, and that so far, that branch of it which seeks after facts, was the only one much cultivated, while the relation of those facts to each other and to causes were as yet untouched. Indeed, semeiology and materia medica stood almost alone. Anatomy, physiology, pathology, and chemistry, were still in the future, and unable to give any light to reason by. The theorists had it all their own way now for some time. The most prominent of them was Asclepiades of Bithynia, who applied to the human body a modification of the atomic theory of Epicurus.

Man, according to him, results from the accidental reunion of atoms which affect a determinate form, and whose movement, regular or irregular in the space assigned to them, constitutes health or disease. One of his principal doctrines was an exaltation of the powers of wine, which he calls a sovereign, incomparable and divine remedy. His disciple, Themison, formed what was called the methodical school,which appears to have been specially remarkable for the extreme formality of its doctrines. He founded everything on the *strictum et laxum*, that is, too great contraction or relaxation. It is curious how in some cases very sensible modes of treatment were deduced from such general doctrines as those of the methodist. For instance, in dropsy, their great object was to effect the restoration of the forces, in order to restore to the skin that tonicity, to the absence of which they attributed the disease. For this purpose they recommended rubefacients, powerful sudorifics, and hot baths, together with purgatives and diuretics. This was pretty good for those ancient parties, considering that they knew nothing of the anatomy of the kidneys, nor the histology of the skin, nor of the vicarious relations which physiology has demonstrated to exist between them, nor of the circulation of the blood. I account for their clear-headed notions of dropsy, by the fact that it is to some extent an external disease, and tells its story pretty plainly by swelling, retention of urine, and so on.

The sect of pneumatists differed from the methodists principally in adding the influence of the "*pneuma*," or spirit, to the other doctrines. They paid particular attention to the mixture of the elements, as they called them, and to the influence of "sensible qualities." According to them, heat and moisture mixed together, are the elements most favorable to health. Heat and dryness occasion acute disease; cold and moisture, phlegmatic disease; cold and dryness give birth to melancholy. Everything dries and becomes cold at the approach of death. To this school belonged Archigines, and Aretaeus, the best observer of antiquity after Hippocrates. He is especially remarkable for the attention which he paid to the forces of nature, the individual differences of the constitutions of men, and the effects of climate on disease. And thus you see that these men who went so far astray in their speculations as to place themselves among the class whom I have called theorists, yet were giving some assistance to medical philosophy by pushing its investigating branch a little farther in a new direction.

The sect of theorists culminated in Galen. It is interesting to note the history of the efforts of that great mind to master the secrets of nature without the aid of the great discoveries and inventions which the philosopher of modern times is armed with. Starting with the premises of the few facts that had been collated before his day, Galen, by applying to everything he studied what we would call in these days "common sense," produced astonishing results in the way of knowledge of disease, and especially of prognosis. He went so far into the ranks of the theorists as to accept the doctrine of the four elements, and to construct theories thereon—but, nevertheless, his great object seems to have been to exercise a true eclecticism by choosing the best theories and most authenticated facts, and to systematize the whole in one grand plan. He made an effort at distinction between matter and force under the names of *elements* and *principles* of bodies; the "principles" meaning that force which we now recognize in its manifestations of heat, light, electricity, etc. Galen was also aware that the union of two elements produced a third, unlike either of the others—the *tertium quid* of chemistry. But he missed it when he taught that there was "excess of fire in the bile and earth in the atrabile, that is, unless the theory of some young electrical enthusiast which I read in a Medical Journal recently, should turn out to be correct. He thinks that the liver is a kind of electrical battery for the brain, and as heat is known to accompany electricity, why it might be expected that if this were the case, the bile would get warmed up a little in the gall bladder. Galen taught that the morbid states of organs depended on the number, size, figure, and situation of the parts, which was good for the imperfect knowledge of his time, but a long way from the grand principle which we can enunciate at *this* day, that disease is a morbid state of *structure* of an organ. To every alteration of the humors, so called, Galen gave the name of putridity. This takes place, he says, every time that a stagnant humor is exposed to a high temperature, without being able to evaporate. This humoral doctrine I am disposed to consider in a humorous light. Are *you?* According to Galen a fever is the result of putridity developing heat, in which the blood vessels take part.

I will not attempt a further sketch of Galen's teachings—it would weary you. He had a good notion of hémorrhagic affections, and the good he did was in studying with intense devotion all the circumstances he could find to influence health and disease. His success was limited by the scantiness of important knowledge, and the means of getting it.

With the decline of the Roman Empire declined philosophy of medicine, sharing the fate of general philosophy. A few incidental discoveries or observations might be made, but all life and energy were gone. Down to the latest periods of the Grecian Empire, medical authors occasionally appeared, but except the suggestion sometimes of new remedies, there is little to attract our attention in looking after philosophy of medicine.

We owe the institution of hospitals in the earlier periods of the Greek Empire, to the influence of the spirit of Christianity. The Crusades both imported new and formidable diseases from the East, and created a spirit which devised the means of combating them. The hospital at Jerusalem for the relief of pilgrims in the Holy Land, founded by merchants of Amalfi, is carried back by some as far as the seventh century.

The formal re-establishment of medicine in Europe dates from the formation of the school at Salerno, about the close of the tenth century. This school had a regular curriculum of studies, and gave certificates of qualification on examination. I apprehend that their course would not compare favorably with the Spring term at Rush, and I suspect that a lecture on philosophy of medicine was not included in their proceedings; but still this was progress, and thus Europe possessed, even during the dark ages, two admirable institutions for the advancement of medicine which hardly existed in ancient times, namely, hospitals, where disease could be easily and extensively studied, and a system of attesting the qualifications of those devoted to the profession.

It was not long before these institutions bore fruit. When all was submission to the authority of the ancients in *general* science, we can trace some independent thinking in medicine. Still the science was subject to the same kind of general influence as other branches of learning. After the revival of letters, Galen was worshiped very much like Aristotle, but the numerous discoveries of the alchemists, the detection of several of the anatomical errors of Galen, and, most of all, the spirit of original thinking which began to arise, prepared the way for modern medicine.

One remarkable man accelerated greatly the approaching revolution, and stands out in bold relief, a portentous figure between the old and the new—the extraordinary Paracelsus. The revival of letters had brought with the doctrines of the ancient philosophers and physicians such an extreme veneration for their authority, that this feeling threatened to chain the human mind for ages. In philosophy, these chains were broken by Bacon and others. Paracelsus had a career as extraordinary as his doctrines. He boasts that at a very early period he had traversed Spain, Portugal, Prussia,

Poland, Hungary, and other countries. He undoubtedly picked up some valuable chemical secrets from the empirics of his time. His doctrines, with the single exception perhaps of that of tartar emetic, which really does him credit, are an absurd combination of the wildest and most extravagant notions regarding the vital spirit, the influence of planetary bodies, etc. The best part of Paracelsus was his knowledge of therapeutics; perhaps you remember his name in connection with the tincture of aloes and myrrh. "The Elixir Proprietatis of Paracelsus."

> "One full-sized bottle stood upon the shelf,
> Which held the medicine that he took himself;
> Whate'er the reason, it must be confessed
> He filled that bottle oftener than the rest;
> What drug it held I don't presume to know,
> The gilded label said "Elixir Pro."

About the sixteenth century, medical men might on the whole be divided into two classes, the fanatical innovators, like Paracelsus, and those who defended the authority of the ancients with equal fanaticism. The lever used by the former was chemistry, and the new remedies which this science was continually making known; hence the battle between the two parties was fought on this ground. Of the almost incredible lengths to which men were led in the controversy between the Chemists and the Galen-ists, we have an example in Guy Patin, who was the zealous opponent of the new doctrine. He has not given us a refutation of chemical medicine, but many proofs of the irreconcilable and blind hatred which he entertained for the chemists of his time. He always speaks of his opponents as malefactors, and doubtless would have treated them as such. He boasts that he never administered an antimonial preparation, and maintains that antimony had slain more men than a long war. His *Martyrologium Antimonii* is a record of the deaths and misfortunes caused, according to him, by the detested drug. At length, in 1666, a formal vote of the faculty of Paris sanctioned the use of antimony.

When, after the long night of barbarism which followed the downfall of the Roman Empire, the flight of the learned Greeks before the conquering Ottomans carried the study of the ancient writers into Europe, the arts had been gradually making great progress, and the effect of the lessons derived from antiquity upon the minds of the energetic nations of the West was very different from what it had been with the slavish and degraded Greeks. Still, for a long time after the revival of letters, the aspect of science was very much as it had been in Greece. The scholastic controversies of the middle ages were passing away, but the doctrines of the ancient sects were professed in the universities of Europe, and Aristotle in particular was almost worshiped, and his works esteemed a sort of bible of science. Great stress in particular was laid on his physics, the worst part of his philosophy. But the alchemist had laid the foundation of an experimental study; several reformers had attacked the doctrines of Aristotle; some experiments had shown the fallacy of his physics, and, above all, the arts had become capable of furnishing real aid to the inquirer. Great observers, like Vesalius and Gilbert, flourished. Then arose Bacon; he proclaimed the omnipotence of common sense; he showed the madness of the dogmatists, the extravagance of the alchemists, and the pedantry of the schools; he boldly attacked the peripatetics or Aristotelians, who occupied nearly all the chairs of the universities; he insisted on the necessity of observation, and especially, he showed the value and power of experiment. He always kept in view the great importance of medicine, and exhorted its cultivators to work in the right direction, and abandon vague speculation and too great reliance on authority.

> "Trust not the frantic or mysterious guide,
> Nor stoop a captive to the schoolmen's pride;
> On nature's wonders fix alone thy zeal,
> They dim not reason."

In considering the share of merit due to Bacon and to the illustrious confederates, who, nearly contemporaneously with him, labored to change the direction of the energies of mankind, the first place is surely due to the immortal author of the Novum Organum. He was the legislator of science. Others joined in the glorious enterprise, but he gave permanence to the efforts of all. In the battle array against the peripatetics, he was the general, though there were others, brave soldiers. In building up the edifice of science, he was as the architect, they the skilled workmen; or, while such as Galileo acted as the grand pioneers who leveled the paths of science and removed the obstacles, Bacon, aloof, as from a lofty tower, planned and directed the march of the intellect.

The views of Lord Bacon on the subject of medicine are peculiarly worthy of our attention, and we may, I think, clearly trace these views in operation in the succeeding age in the language and the actions of the true restorer of Hippocratic medicine, the great Sydenham. Bacon's views on the subject will be found principally in his *De Augmentis Scientiarum*, but they are scattered through all his works, and his philosophy is now felt in all the departments of medicine, as well as other sciences.

Bacon states medicine to be necessarily a somewhat conjectural art, because it has to deal with a subject extremely complicated, and subject to many variations. He deplores the want of fidelity and attention on the part of observers, who ought to endeavor to present correct pictures of disease, instead of attaching such value to opinions and hypothesis. Accounts of disease should not be too prolix, mentioning things of no consequence and of daily occurrence, neither should they be too brief and meagre. In fact, many things not new in themselves, may be observed in new lights, so that descriptions should not be confined to extraordinary events.

Bacon particularizes the neglect of morbid anatomy in his time, and the exclusive tendency to a humoral pathology which prevailed. He exhorts medical men to a more particular study of the diseases reputed incurable, so as to try and find out more powerful means of cure, and thus diminish the number of quacks—a piece of advice which is not without value at present. He complains of the want of real power in the medicine of his day, and attributes the success of quacks to this cause. Perhaps those who formed themselves on the model of Bacon, entertained too great a repugnance for speculation; but, especially in that age, this was the safe error. As we can attribute the spirit which produced a Harvey, a Locke, and a Boyle, to the direct influence of the writings of Bacon, whose followers threw themselves on the vast field of science indicated by the author of the *Novum Organum*, as the Spaniards in the age after Columbus rushed on the new world, so we may reasonably attribute to the same influence, the peculiar character which the genius of Sydenham assumed. The following passages show how thoroughly Sydenham was imbued with the spirit of the Baconian philosophy.

He says: "As truly as the physician may collect points of diagnosis from the minutest circumstance of the disease, so truly may he elicit indications in the way of therapeutics. So much does this statement hold good, that I have often thought that, provided with a thorough insight into the history of any disease whatsoever, I could invariably apply an equivalent remedy. A clear path being thus marked out for me by the different phenomena of the complaint, these phenomena, if carefully collated with each other, lead as it were by the hand, to those palpable indications of treatment which were drawn, not from the hallucinations of our fancy, but from the innermost penetralia of nature."

Sydenham continues: "I am far from denying that the physician ought to attend diligently to particular cases in respect to the results both of the methods and of the remedies which he employs in the cure of disease. I grant, too, that he may lay up his experience for use, both in the way of easing his memory, and seizing suggestions. By so doing, he may gradually increase in medical skill, so that eventually by long continuance and frequent repetition of his experiments, he may lay down and prescribe for himself a method from which, in the case of this or that disease, he need not deviate a single straw's breadth. Nevertheless, the publication of particular observations is in my mind of no great advantage. Where is the particular importance in just telling us that once, twice, or even oftener, this disease has yielded to that remedy. We are overwhelmed, as it *is*, with infinite abundance of vaunted mediciments, and here they add a new one. Now, if I repudiate the rest of my formula and restrict myself to this medicine only, I must try its efficacy by innumerable experiments, and I must weigh in respect to both the patient and the practice, innumerable circumstances, before I can derive any benefit from such a solitary observation."

Such are the words of the great Sydenham. Given in plain American, they simply mean that when he knows exactly what's the matter, he finds little difficulty in treating the disease. In this spirit of medical philosophy must every one be content to labor, who is really desirous of advancing his profession, and earning a durable reputation.

Far different has been the method pursued by empirics and greedy speculators on the misery and weakness of mankind. Their object is not to advance truth, but to acquire the name of curing disease, and *this* is within the power of any one, either in the profession or out of it, who is sufficiently bold and unscrupulous.

> From powerful causes spring the empiric's gains,
> Man's love of life, his weakness and his pains ;
> These first induce him the vile trash to try,
> Then lend his name, that other men may buy ;
> This law of life, which in our nature rules,
> To *vile imposture* makes us dupes and tools ;
> Then pain compels th' impatient soul to seize,
> On *promised* hopes of instantaneous ease ;
> And weakness, too, with every wish complies,
> Worn out and won by *importunities.*

